# Phase II randomised study of magrolimab combined with bevacizumab-FOLFIRI in patients with previously treated advanced inoperable metastatic colorectal cancer

**DOI:** 10.1016/j.esmogo.2026.100324

**Published:** 2026-04-17

**Authors:** M. Fakih, S. Gill, K. Sampat, D.L. Chan, G. Fisher, M. Cruz-Correa, H.-J. Lenz, P. Garcia-Alfonso, F. Forget, S. Lonardi, J. Krauss, E. Chen, A. Cubillo Gracian, Y. Liu, N. van Buuren, J. Shao, M. Dong, A. Chen, M. Tejani, J.R. Hecht

**Affiliations:** 1 City of Hope Comprehensive Cancer Center, Duarte, Australia; 2 Alfred Health, Melbourne, Australia; 3 Virginia Cancer Specialists PC, Arlington, USA; 4 University of Sydney, Camperdown, Australia; 5 Stanford Cancer Center, Palo Alto, USA; 6 PanOncology Trials (Pan American Center for Oncology Trials, LLC), San Juan, USA; 7 USC Norris Comprehensive Cancer Center, Los Angeles, USA; 8 Hospital General Universitario Gregorio Maranon, Madrid, Spain; 9 Centre Hospitalizer De L’Ardenne, Libramont-Chevigny, Belgium; 10 Istituto Oncologico Veneto IRCCS, Padova, Italy; 11 University of Michigan, Ann Arbor, USA; 12 Princess Margaret Cancer Centre, Toronto, Canada; 13 Hospital HM Sanchinarro, Madrid, Spain; 14 Gilead Sciences, Inc., Foster City, USA; 15 AdventHealth, Orlando, USA; 16 University of California Los Angeles (UCLA), Santa Monica, USA

**Keywords:** metastatic colorectal cancer, magrolimab, bevacizumab, FOLFIRI

## Abstract

**Background:**

Effective treatment for metastatic colorectal cancer (mCRC) remains an unmet need. CD47 overexpression on mCRC cells is associated with a poor prognosis. The ELEVATE CRC study evaluated magrolimab (CD47 inhibitor) plus bevacizumab-5-fluorouracil, irinotecan and leucovorin (FOLFIRI) in mCRC.

**Patients and methods:**

This open-label, phase II study enrolled eligible patients with previously treated advanced inoperable mCRC. The safety run-in (SRI) cohort’s primary objective was to evaluate the safety, tolerability and recommended phase II dose of magrolimab plus bevacizumab-FOLFIRI. After SRI, patients were randomly assigned (2:1) to receive magrolimab plus bevacizumab-FOLFIRI (magrolimab) or bevacizumab-FOLFIRI (control). The randomised cohort’s primary endpoint was progression-free survival (PFS).

**Results:**

No dose-limiting toxicities occurred in the SRI cohort (10 patients). In the randomised cohort (67 patients), median PFS was 6.2 versus 6.7 months in the magrolimab (44 patients) versus control (23 patients) arm (hazard ratio 0.956, 95% confidence interval 0.339-2.691). However, early study closure limits PFS interpretation. Grade ≥3 adverse event (AE) rates were 72.7% versus 52.4% in magrolimab versus control arms. Across cohorts, two AE-related deaths occurred in patients treated with magrolimab; neither was considered treatment-related.

**Conclusions:**

Adding magrolimab to bevacizumab-FOLFIRI increased grade ≥3 AE incidence and did not improve efficacy outcomes, highlighting a continuing unmet therapeutic need in mCRC.

## Highlights

• This phase II study tested the safety and efficacy of magrolimab combined with bevacizumab-FOLFIRI in mCRC.
• Adding magrolimab to bevacizumab-FOLFIRI produced a median PFS similar to bevacizumab-FOLFIRI alone (6.2 versus 6.7 months).
• Addition of magrolimab to bevacizumab-FOLFIRI increased grade ≥3 AE rates with no new safety signals.

## Introduction

Metastatic colorectal cancer (mCRC) has a poor prognosis with an ≈15% 5-year survival rate.^1^ Although immune checkpoint inhibitors and targeted therapy have improved outcomes in patients with certain mutational profiles (e.g. pembrolizumab for high microsatellite instability), most patients with mCRC are ineligible for or do not respond to these treatments.^2^ For those patients, bevacizumab combined with fluoropyrimidine-based chemotherapy [e.g. 5-fluorouracil (5-FU)], irinotecan and leucovorin (FOLFIRI)] is considered one standard treatment in first- and second-line settings.^2^ However, responses and survival observed with this combination in the second line are limited [partial response (PR) 11%, progression-free survival (PFS) <6.5 months^3^]; there remains a need for novel agents to improve treatment efficacy with acceptable toxicity.

Magrolimab is a monoclonal antibody that targets cluster-of-differentiation 47 (CD47), which acts, in part, as a signal preventing phagocytosis.^4^^,^^5^ CD47 is overexpressed in many tumour types and associated with a poor prognosis in CRC.^6^ In preclinical models of solid tumours (including colon) and haematological malignancies, magrolimab treatment triggered macrophage-mediated cancer cell phagocytosis and tumour growth inhibition.^4^^,^^5^ Furthermore, combining bevacizumab,^7^ 5-FU^8^ or irinotecan^9^ with an anti-CD47 agent has demonstrated synergistic antitumour activity in preclinical models of CRC and gastric cancer. This may be due to the ability of bevacizumab and chemotherapeutics to modulate the tumour microenvironment and/or induce pro-phagocytic signals.^9^^,^^10^ Building on these preclinical data, a prior phase Ib/II clinical study in advanced CRC and other solid tumours showed that magrolimab had an acceptable safety profile up to a maintenance dose of 45 mg/kg (combined with cetuximab).^11^ In this article, we report results from the randomised, phase II ELEVATE CRC study, which was designed to evaluate the safety and efficacy of magrolimab plus bevacizumab and FOLFIRI in previously treated, advanced mCRC.

## Patients and methods

### Study design and treatments

This phase II, randomised, open-label, multicentre study (NCT05330429) enrolled adults (aged ≥18 years) with histologically/cytologically confirmed adenocarcinoma originating in the colon or rectum (excluding appendiceal and anal canal cancers). Eligible patients had disease progression on or after one prior systemic therapy (chemotherapy based on 5-FU or capecitabine with oxaliplatin and either bevacizumab, or for patients with *RAS* wild-type and left-sided tumours, bevacizumab, cetuximab or panitumumab) wherein curative resection was not indicated. Only patients ineligible for immune checkpoint inhibitor treatment (i.e. tumours lacking high microsatellite instability or mismatch repair deficiency) and without a *BRAF V600E* mutation were enrolled. Other key eligibility criteria included measurable disease per Response Evaluation Criteria in Solid Tumours, version 1.1 (RECIST v1.1), and an Eastern Cooperative Oncology Group performance status 0 or 1. Additional inclusion/exclusion criteria are summarised in the Supplementary Methods, available at https://doi.org/10.1016/j.esmogo.2026.100324.

A safety run-in (SRI) cohort followed by a randomised cohort was planned. In the SRI cohort, patients received magrolimab plus bevacizumab-FOLFIRI to determine a magrolimab recommended phase II dose (RP2D) in this combination. Later, patients were randomly assigned in a 2:1 ratio to receive magrolimab plus bevacizumab-FOLFIRI (magrolimab arm) or bevacizumab-FOLFIRI alone (control) as per the dosing schedule (Supplementary Table S1, available at https://doi.org/10.1016/j.esmogo.2026.100324). Randomisation stratification factors were KRAS receptor mutation/unknown versus wild-type, geographic region (United States versus ex-United States) and presence versus absence of liver metastases. The study was conducted according to the International Conference on Harmonisation Good Clinical Practice Guidelines, the Declaration of Helsinki and local institutional review board requirements. All patients provided written informed consent before study participation.

### Outcomes

Primary endpoints for the SRI cohort were incidence of dose-limiting toxicities (DLTs), treatment-emergent adverse events (TEAEs) and laboratory abnormalities per the National Cancer Institute Common Terminology Criteria for Adverse Events version 5.0 (Supplementary Methods, available at https://doi.org/10.1016/j.esmogo.2026.100324). The randomised cohort’s primary endpoint was PFS (time from randomisation until investigator-assessed disease progression per RECIST v1.1, or death from any cause, whichever occurs first). Secondary endpoints for the randomised cohort included confirmed objective response rate (ORR; proportion of patients with a complete response or PR on two assessments, at least 28 days apart) per investigator using RECIST v1.1, duration of response (DOR; time from first objective response until disease progression or death from any cause, whichever occurs first) and overall survival (OS; time from randomisation to death from any cause). Assessment of the correlation between clinical response [ORR and disease control rate (DCR)] and baseline tumour cell membrane CD47 levels was exploratory (Supplementary Methods, available at https://doi.org/10.1016/j.esmogo.2026.100324).

### Statistical analysis

Planned enrolment was 6-18 patients in the SRI cohort and ≈117 patients in the randomised cohort (magrolimab arm, *n* = 78; control arm, *n* = 39; Supplementary Methods, available at https://doi.org/10.1016/j.esmogo.2026.100324). The study was closed early before the planned number of patients had been randomly allocated to the study (67 of 117 planned).

The DLT-assessable population included patients enrolled in the SRI cohort who completed the 28-day DLT assessment period and received ≥3 doses of magrolimab and ≥2 doses of bevacizumab and FOLFIRI. Safety was assessed in patients who received ≥1 dose of any study drug in either cohort.

For the randomised cohort, efficacy analyses included all patients randomly allocated to the study. PFS, OS and DOR were estimated using the Kaplan-Meier method; for PFS and OS, treatments were compared using the log-rank testing and Cox proportional hazards regression to estimate hazard ratios (HRs) and 2-sided 95% confidence intervals (CIs). For ORR, 95% CIs were based on the Clopper–Pearson exact method. Due to early study closure, efficacy endpoints were not stratified by randomisation factors. Descriptive statistics were used to summarise all other outcomes.

## Results

### Patients

At data extraction (26 July 2024), a total of 77 patients had enrolled, with 10 in the SRI cohort, 44 randomly assigned to the magrolimab arm and 23 randomly assigned to the control arm (Supplementary Figure S1, available at https://doi.org/10.1016/j.esmogo.2026.100324). Two patients randomly assigned to the control arm did not receive treatment. Baseline demographics and characteristics are summarised in Table 1. In the magrolimab and control arms, most patients had received at least one prior regimen in the metastatic setting (95.5% and 95.7%), were KRAS receptor mutation-positive/unknown (68.2% and 69.6%) and had liver metastases (72.7% and 69.6%). The most common reason for study discontinuation across treatment arms was study closure (Supplementary Figure S1, available at https://doi.org/10.1016/j.esmogo.2026.100324). Protocol deviations are listed in Supplementary Table S2, available at https://doi.org/10.1016/j.esmogo.2026.100324.

**Table 1:** Patient demographics and baseline disease characteristics

	Magrolimab + bevacizumab-FOLFIRI			Bevacizumab-FOLFIRI
	SRI cohort (*n* = 10)	Randomised cohort (*n* = 44)	Combined (*n* = 54)	Randomised cohort (*n* = 23)
Age, median (range), years	53 (31-66)	59 (34-77)	58 (31-77)	56 (33-81)
Male, *n* (%)	4 (40.0)	27 (61.4)	31 (57.4)	14 (60.9)
Race, *n* (%)				
Asian	0	5 (11.4)	5 (9.3)	2 (8.7)
Black or African American	1 (10.0)	4 (9.1)	5 (9.3)	1 (4.3)
White	9 (90.0)	31 (70.5)	40 (74.1)	19 (82.6)
Other/not permitted^a^	0	4 (9.1)	4 (7.4)	1 (4.3)
Geographic region,^b^*n* (%)				
United States	NA	20 (45.5)	20 (37.0)	11 (47.8)
Ex-United States	NA	24 (54.5)	24 (44.4)	12 (52.2)
ECOG PS,^c^*n* (%)				
0	7 (70.0)	29 (65.9)	36 (66.7)	15 (65.2)
1	3 (30.0)	15 (34.1)	18 (33.3)	6 (26.1)
KRAS receptor status,^b^*n* (%)				
Mutation/unknown	NA	30 (68.2)	30 (55.6)	16 (69.6)
Wild-type	NA	14 (31.8)	14 (25.9)	7 (30.4)
Liver metastases present,^b^*n* (%)	NA	32 (72.7)	32 (59.3)	16 (69.6)
No. of prior regimens in any setting, *n* (%)				
1	7 (70.0)	38 (86.4)	45 (83.3)	20 (87.0)
2	3 (30.0)	5 (11.4)	8 (14.8)	2 (8.7)
4	0	1 (2.3)	1 (1.9)	1 (4.3)
No. of prior lines in the metastatic setting, *n* (%)				
1	8 (80.0)	41 (93.2)	49 (90.7)	20 (87.0)
2	2 (20.0)	0	2 (3.7)	2 (8.7)
4	0	1 (2.3)	1 (1.9)	0

Data presented for all enrolled patients.

ECOG PS, Eastern Cooperative Oncology Group performance status; ex-United States, outside the United States; FOLFIRI, folinic acid (leucovorin), 5-fluorouracil and irinotecan; IXRS, interactive response system; KRAS, Kirsten rat sarcoma; NA, not applicable; SRI, safety run-in.

a Could not be provided due to regulations or if the patient refused to disclose.

b Assessed by IXRS in the randomised cohort only.

c Missing: bevacizumab-FOLFIRI, *n* = 2 (8.7%).

Following discontinuation of magrolimab development in haematological malignancies, a strategic decision was made by the sponsor to end further development of magrolimab. Study closure was not based on any safety concerns or futility analyses conducted.

### SRI results

No DLTs occurred in the SRI cohort (*n* = 10). Any-grade and grade ≥3 TEAEs were experienced by 10 (100%) and 9 (90.0%) patients, respectively (Table 2). The most common grade ≥3 TEAE was neutropenia (*n* = 6; 60.0%). TEAEs of clinical importance are summarised in Supplementary Table S3, available at https://doi.org/10.1016/j.esmogo.2026.100324. TEAEs led to discontinuation of any study drug in two (20.0%) patients and dose reduction of any study drug in six (60.0%) patients. One death occurred due to a TEAE (sepsis), which was not considered to be related to study treatment. The RP2D for magrolimab was determined to be 30 mg/kg.

**Table 2:** Most common TEAEs

TEAE, *n* (%)	Magrolimab + bevacizumab-FOLFIRI						Bevacizumab-FOLFIRI	
	SRI cohort (*n* = 10)		Randomised cohort (*n* = 44)		Combined (*n* = 54)		Randomised cohort (*n* = 21)	
	Any grade	Grade ≥3	Any grade	Grade ≥3	Any grade	Grade ≥3	Any grade	Grade ≥3
Total	10 (100)	9 (90.0)	44 (100)	32 (72.7)	54 (100)	41 (75.9)	19 (90.5)	11 (52.4)
Related to any study drug	10 (100)	7 (70.0)	44 (100)	30 (68.2)	54 (100)	37 (68.5)	18 (85.7)	9 (42.9)
Nausea	9 (90.0)	0	23 (52.3)	0	32 (59.3)	0	10 (47.6)	0
Anaemia	6 (60.0)	1 (10.0)	25 (56.8)	12 (27.3)	31 (57.4)	13 (24.1)	4 (19.0)	0
Diarrhoea	6 (60.0)	0	25 (56.8)	3 (6.8)	31 (57.4)	3 (5.6)	11 (52.4)	0
Neutropenia^a^	7 (70.0)	6 (60.0)	23 (52.3)	16 (36.4)	30 (55.6)	22 (40.7)	10 (47.6)	7 (33.3)
Fatigue	8 (80.0)	0	19 (43.2)	1 (2.3)	27 (50.0)	1 (1.9)	6 (28.6)	1 (4.8)
Stomatitis	5 (50.0)	0	16 (36.4)	3 (6.8)	21 (38.9)	3 (5.6)	2 (9.5)	1 (4.8)
Decreased appetite	4 (40.0)	0	15 (34.1)	0	19 (35.2)	0	6 (28.6)	1 (4.8)
Headache	4 (40.0)	0	15 (34.1)	0	19 (35.2)	0	0	0
Vomiting	4 (40.0)	1 (10.0)	14 (31.8)	1 (2.3)	18 (33.3)	2 (3.7)	6 (28.6)	1 (4.8)
Thrombocytopenia^b^	5 (50.0)	1 (10.0)	11 (25.0)	0	16 (29.6)	1 (1.9)	1 (4.8)	0
Constipation	4 (40.0)	0	9 (20.5)	0	13 (24.1)	0	5 (23.8)	0
Abdominal pain	2 (20.0)	0	10 (22.7)	1 (2.3)	12 (22.2)	1 (1.9)	4 (19.0)	1 (4.8)
Alopecia	1 (10.0)	0	9 (20.5)	0	10 (18.5)	0	7 (33.3)	0
Pyrexia	3 (30.0)	0	7 (15.9)	0	10 (18.5)	0	4 (19.0)	0
WBC decreased	1 (10.0)	0	9 (20.5)	6 (13.6)	10 (18.5)	6 (11.1)	2 (9.5)	1 (4.8)
Asthenia	0	0	9 (20.5)	0	9 (16.7)	0	3 (14.3)	0
Infusion-related reaction	4 (40.0)	0	5 (11.4)	1 (2.3)	9 (16.7)	1 (1.9)	0	0
Epistaxis	1 (10.0)	0	6 (13.6)	0	7 (13.0)	0	5 (23.8)	0
Weight decreased	1 (10.0)	0	6 (13.6)	0	7 (13.0)	0	4 (19.0)	0
Dyspnoea	0	0	5 (11.4)	0	5 (9.3)	0	5 (23.8)	0

AEs were coded according to the Medical Dictionary for Regulatory Activities, version 27.0. Multiple AEs were counted only once per patient for the highest severity grade for each preferred term. Any-grade AEs with incidence ≥15% in either combined magrolimab + bevacizumab-FOLFIRI or randomised bevacizumab-FOLFIRI treatment groups are presented with corresponding grade ≥3 AEs.

AE, adverse event; FOLFIRI, folinic acid (leucovorin), 5-fluorouracil and irinotecan; SRI, safety run-in; TEAE, treatment-emergent adverse event; WBC, white blood cell.

Data presented for all patients who received ≥1 dose of any study drug.

a Grouped terms of neutropenia and neutrophil count decreased.

b Grouped terms of thrombocytopenia and platelet count decreased.

### Randomised cohort: efficacy

**Figure 1:**
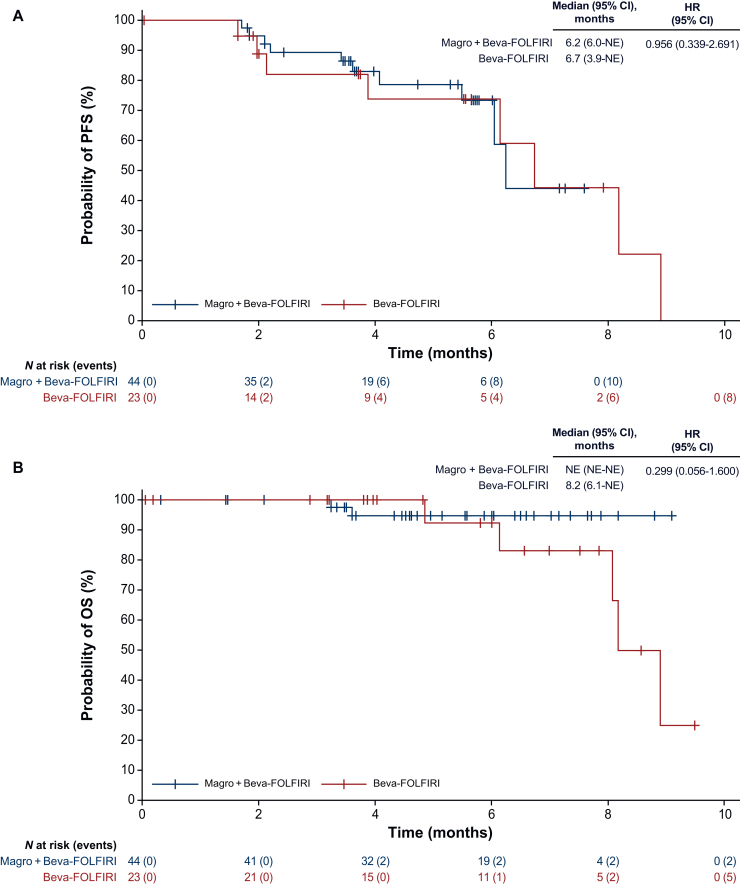
**Survival outcomes.** (A) Progression-free survival. (B) Overall survival. Beva-FOLFIRI, bevacizumab plus folinic acid (leucovorin), 5-fluorouracil and irinotecan; CI, confidence interval; HR, hazard ratio; Magro, magrolimab; NE, not estimable; OS, overall survival; PFS, progression-free survival.

The median PFS was 6.2 months in the magrolimab arm versus 6.7 months in the control arm (HR 0.956, 95% CI 0.339-2.691; Figure 1A); median OS was not estimable versus 8.2 months (HR 0.299, 95% CI 0.056-1.600; Figure 1B). No responses were observed in the control arm. In the magrolimab arm, the confirmed ORR was 13.6% (all PRs); median DOR and time to response were not estimable and 2.1 months, respectively (Table 3).

**Table 3:** Response outcomes in patients randomly allocated to the study

	Magrolimab + bevacizumab-FOLFIRI (*n* = 44)	Bevacizumab-FOLFIRI (*n* = 23)
ORR^a^^,^^b^^,^^c^ (95% CI), %	13.6 (5.2-27.4)	0 (0-14.8)
Odds ratio (95% CI)	NE (NE-NE)	
CR, *n* (%)	0	0
PR, *n* (%)	6 (13.6)	0
SD, *n* (%)	28 (63.6)	16 (69.6)
PD, *n* (%)	5 (11.4)	3 (13.0)
DCR (95% CI), %	77.3 (62.2-88.5)	69.6 (47.1-86.8)
Median time to response (range), months	2.1 (1.8-3.7)	NA
Median DOR^a^ (95% CI), months	NE (NE-NE)	NA

Data presented for all enrolled patients.

CI, confidence interval; CR, complete response; DCR, disease control rate; DOR, duration of response; FOLFIRI, folinic acid (leucovorin), 5-fluorouracil and irinotecan; NA, not applicable; NE, not evaluable; ORR, objective response rate; PD, progressive disease; PR, partial response; RECIST, Response Evaluation Criteria in Solid Tumours; SD, stable disease.

a Investigator-assessed per RECIST v1.1.

b Confirmed.

c Discontinued before reaching first assessment: magrolimab + bevacizumab-FOLFIRI, 5 (11.4%); bevacizumab-FOLFIRI, 4 (17.4%).

### Randomised cohort: safety

The median duration of exposure to study drugs was similar (median cycles, 4.0) (Supplementary Table S4, available at https://doi.org/10.1016/j.esmogo.2026.100324). The most common types of TEAEs observed are summarised in Table 2. Grade ≥3 TEAE rates were 72.7% in the magrolimab arm and 52.4% in the control arm; serious TEAE rates were 36.4% and 28.6%. The most common grade ≥3 TEAE in both treatment arms was neutropenia (36.4% and 33.3%, respectively). Regarding TEAEs of clinical importance in magrolimab versus control arms, anaemia rates were 59.1% versus 19.0%, pneumonitis rates were 6.8% versus 0% and serious infection rates were 13.6% versus 4.8% (Supplementary Table S3, available at https://doi.org/10.1016/j.esmogo.2026.100324).

TEAEs led to discontinuation of any study drug in six (13.6%) patients in the magrolimab arm and two (9.5%) patients in the control arm. TEAEs led to dose reduction of any study drug in 17 (38.6%) and 10 (47.6%) patients, respectively. One death occurred due to a TEAE [coronavirus disease 2019 (COVID-19)] in the magrolimab arm and was not considered related to any study treatment. The magrolimab combination safety profile was consistent across the SRI and randomised cohorts (Table 3).

### Exploratory biomarker analysis

In biomarker-assessable patients, DCR and ORR were numerically higher for those treated with magrolimab plus bevacizumab-FOLFIRI (SRI + randomised cohorts, *n* = 44) who had high (≥20%) versus low (<20%) CD47 positivity at baseline; the opposite was observed for DCR among patients given bevacizumab-FOLFIRI (randomised cohort, *n* = 18) (Supplementary Figure S2, available at https://doi.org/10.1016/j.esmogo.2026.100324). PFS was similar for high versus low baseline CD47 expression regardless of treatment type (data not shown). Baseline characteristics and efficacy between biomarker-assessable patients and the intention-to-treat population were similar.

## Discussion

In this study, magrolimab plus bevacizumab-FOLFIRI had no DLTs. Across cohorts, the types of TEAEs observed with magrolimab plus bevacizumab-FOLFIRI in CRC were consistent with those in previous studies.^3^^,^^11^, ^12^, ^13^, ^14^ Adding magrolimab to bevacizumab-FOLFIRI resulted in a higher frequency of grade ≥3 and serious TEAEs; however, due to early study closure and small sample sizes, results should be interpreted with caution.

Anaemia, a known on-target effect of magrolimab, likely contributed substantially to the between-treatment rate disparity for serious TEAEs in ELEVATE CRC. Although timing was not evaluated, results of previous studies have demonstrated that magrolimab-associated anaemia events typically occur early in the treatment course (within ≈2 months) and are uncommon thereafter.^13^^,^^14^^,^^15^ Magrolimab has been associated with an increased incidence of fatal infections versus standard of care in acute myeloid leukaemia/myelodysplastic syndromes.^16^^,^^17^ However, the exact mechanism remains unknown as these haematological malignancies predispose patients to haematological adverse events and, consequently, infection. In this study, serious infection rates differed between treatments, but small sample sizes limit interpretation.

The randomised cohort was designed to evaluate the efficacy of magrolimab plus bevacizumab-FOLFIRI in mCRC. Early study closure limited robust statistical evaluation due to the short follow-up period, lack of survival follow-up data and a smaller sample size than specified per protocol. These factors potentially affected the bias, precision and validity of efficacy results because they increased sensitivity to random variation, reduced statistical power and limited endpoint maturity. Therefore, the survival HRs and CIs reported should be interpreted cautiously. Descriptively, the observed PFS at study closure was similar between treatments and consistent with results of previous studies with bevacizumab-FOLFIRI in this setting.^3^ A numerically higher ORR was achieved by adding magrolimab to bevacizumab-FOLFIRI, but these results should be interpreted with caution given the study’s limitations. The unusual 0% ORR observed here for bevacizumab-FOLFIRI was potentially due to the small sample size, differences in investigator reporting given the open-label design or unmeasured confounding factors.

Despite the early closure of ELEVATE CRC and discontinuation of magrolimab development, CD47 remains a target of interest in CRC. A phase II study (NCT05382442) of first-line ligufalimab (anti-CD47 antibody) combined with ivonescimab plus 5-FU, oxaliplatin, irinotecan and leucovorin in mCRC is ongoing. Preliminary results have shown encouraging antitumour activity and safety^18^; however, ligufalimab’s contribution to the combination’s efficacy is unclear, and a randomised phase III trial of first-line ivonescimab monotherapy has begun enrolment.^19^ A recent, retrospective molecular profiling study showed that CD47 expression was associated with activation of major oncogenic signalling pathways, immune-cell infiltration and clinical outcomes in CRC.^20^ Our study also showed a trend between baseline CD47 expression and response rates but not PFS. In the magrolimab arm, patients with high tumour baseline CD47 levels had numerically increased ORR and DCR compared with patients with low tumour baseline CD47 levels. An opposite trend for DCR was observed in the control arm. However, the small sample size and early study closure limit this interpretation. Although these clinical and biomarker data highlight CD47 as a continued target and biomarker of interest, additional studies are needed to help elucidate the optimal application of anti-CD47-based therapy and the potential role of CD47 as a predictive biomarker in CRC.

### Conclusions

Although magrolimab development has been discontinued, the ELEVATE CRC results provide important insights into the efficacy and safety of combining an anti-CD47 therapy with a standard treatment in patients with mCRC. These results will inform the development of other anti-CD47 agents for solid tumours, including mCRC. Ultimately, these data reinforce the continued need for novel therapies and drug targets for this challenging disease.
