# Supplementary material for: Phase II randomised study of magrolimab combined with bevacizumab-FOLFIRI in patients with previously treated advanced inoperable metastatic colorectal cancer
Source: ESMO Gastrointest Oncol. 2026 Apr 17;12:100324. doi: 10.1016/j.esmogo.2026.100324 (PMC13098452; doi:10.1016/j.esmogo.2026.100324)
Supplement: Supplementary Methods [file mmc1.docx]

**Phase 2 randomised study of magrolimab combined with bevacizumab-FOLFIRI in patients with previously treated advanced inoperable metastatic colorectal cancer**

M. Fakih, S. Gill, K. Sampat, et al.

**SUPPLEMENT**

**Supplementary Methods**

***Inclusion criteria***

All of the following inclusion criteria must have been met for patients to be eligible for participation:

1. Patient must have provided informed consent and was willing and able to comply with clinic visits and procedures as outlined in the study protocol.
2. Male or female, at least 18 years of age.
3. Previously treated patients with inoperable metastatic colorectal cancer (mCRC) who had progressed on or after 1 prior systemic therapy and who were ineligible for checkpoint inhibitor therapy (patients eligible for checkpoint inhibitor therapy were defined as having high microsatellite instability or mismatch repair deficiency and were excluded).
   1. Maintenance therapies were not counted as separate lines of therapy.
4. Chemotherapy and bevacizumab (if applicable)-free interval of ≥3 weeks.
5. Histologically or cytologically confirmed adenocarcinoma originating in the colon or rectum (excluding appendiceal and anal canal cancers) that had progressed on or after 1 prior systemic therapy in the setting where curative resection is not indicated. This therapy must have included chemotherapy based on 5-FU or capecitabine with oxaliplatin and either bevacizumab, or for patients with *RAS* wild-type and left-sided tumours, bevacizumab, cetuximab or panitumumab.
6. Measurable disease, defined as ≥1 measurable metastatic lesion by Response Evaluation Criteria in Solid Tumours, version 1.1, criteria, with lesion not located in a previous field of radiation. Previously irradiated lesions were considered as measurable disease only if disease progression was unequivocally documented at that site since radiation.
7. An Eastern Cooperative Oncology Group performance status of 0 or 1.
8. A life expectancy of ≥12 weeks.
9. Adequate liver and kidney function.
   1. Patients with known active or chronic hepatitis B or C virus (HBV or HCV) infection were excluded. Patients with HBV or HCV who were not on antiviral therapy and had an undetectable viral load within 3 months before inclusion were potentially eligible. Patients with serologic evidence of prior vaccination to HBV (ie, hepatitis B surface antigen-negative and antibody against hepatitis B surface antigen-positive) were allowed to participate.
10. Adequate cardiac function, without history of:
    1. Hypertensive crisis or hypertensive encephalopathy.
    2. Thromboembolic event (except peripheral deep vein thrombosis treated with anticoagulants) or clinically significant coronary artery disease or myocardial infarction within 6 months before inclusion.
    3. Haemolytic anaemia, autoimmune thrombocytopenia or Evans syndrome within 3 months before inclusion.
    4. Known inherited or acquired bleeding disorders.
11. Haemoglobin levels ≥9 g/dL before the initial dose of study treatment.
    1. Red blood cell (RBC) transfusions were allowed to meet this criterion; however, patients who were RBC transfusion dependent, defined as requiring >2 units of packed RBC transfusions during the 4-week period before screening, were excluded.
12. An absolute neutrophil count ≥1.5 × 10^9^/L.
13. A platelet count ≥100 × 10^9^/L.
14. Completion of pre-treatment blood cross-match.

***Exclusion criteria***

Patients who met any of the exclusion criteria were not allowed to enrol:

1. Patients harbouring known *BRAFV600E* or high microsatellite instability mutations or mismatch repair deficiency.
2. Prior anticancer therapy, including chemotherapy, hormonal therapy or investigational agents, within 3 weeks or within at least 4 half-lives before magrolimab dosing (up to a maximum of 4 weeks), whichever was shorter.
   1. Localised non-central nervous system (CNS) radiotherapy, previous therapy with luteinizing hormone-releasing agonists for breast cancer and treatment with bisphosphonates and receptor activator of nuclear factor κB ligand inhibitors were not criteria for exclusion. There was no required minimum washout period for these therapies; however, patients were required to have recovered from the effects of radiation.
3. Patients who received a dose-escalation scheme (eg, leucovorin/5-FU or capecitabine with bevacizumab followed by 5-FU, oxaliplatin/irinotecan and leucovorin or capecitabine plus oxaliplatin with bevacizumab) and/or who were not bevacizumab tolerant.
4. Patients with prior treatment with irinotecan or cluster of differentiation (CD) 47 or signal regulatory protein α-targeting agents.
5. Known dihydropyrimidine dehydrogenase deficiency or hypersensitivity to any of the study drugs, the metabolites or formulation excipient, including known allergy or hypersensitivity to monoclonal antibodies (bevacizumab, magrolimab), or to Chinese hamster ovarian cell products or any other humanised or recombinant antibodies or any other chemotherapies under study, and their excipients.
6. Second malignancy, except treated basal cell or localised squamous skin carcinomas, localised prostate cancer or other malignancies for which patients were not on active anti-cancer therapies and who were in complete remission for >2 years.
7. Active CNS disease. Patients with asymptomatic and stable, treated CNS lesions (radiation and/or surgery and/or other CNS-directed therapy who had not received corticosteroids for at least 4 weeks) were allowed.
8. Significant disease or medical conditions, as assessed by the investigator and sponsor, that would have substantially increased the risk-benefit ratio of participating in the study, including, but not limited to:
   1. Peripheral neuropathy of grade >2 (per Common Terminology Criteria for Adverse Events, version 5.0 [CTCAE v5.0]).
   2. Persistent grade ≥2 gastrointestinal bleeding.
   3. Acute intestinal obstruction or sub-obstruction, history of inflammatory intestinal disease or extended resection of the small intestine. Presence of a colonic prosthesis.
   4. Unhealed wound, active gastric or duodenal ulcer or bone fracture.
   5. History of abdominal fistulas, trachea-oesophageal fistulas, any other grade 4 gastrointestinal perforations, non-gastrointestinal fistulas or intra-abdominal abscesses 6 months before screening.
   6. Uncontrolled pleural effusion.
   7. Uncontrolled hypercalcaemia (grade ≥2) or symptomatic hypercalcaemia requiring continued use of bisphosphonate therapy.
   8. Uncontrolled tumour-related pain.
   9. Severe/serious systemic infection within 4 weeks of randomisation or any active, uncontrolled infection requiring systemic therapy within 7 days of randomisation.
   10. Presence of a detectable HIV viral load in patients with a known history of the virus.
9. Positive serum pregnancy test or breastfeeding woman.
10. Current participation in another interventional clinical study.

***Adverse events (AEs) and Dose-limiting toxicities (DLTs)***

AEs were summarized on the basis of the date of onset for the event. Treatment-emergent AEs were defined as any AE that began on or after the date of first dose of study drug up to the date of last dose of study treatment +30 days or the day before initiation of subsequent anti-cancer therapy, whichever comes first. All toxicities were graded according to CTCAE v5.0.

A DLT was defined as any:

- Grade ≥3 haematologic toxicity, including
  - Grade 3 haemolytic anaemia that was medically significant, requiring hospitalization or prolongation of existing hospitalization, disabling or limiting self-care activities of daily life.
  - Grade 4 neutropenia lasting >7 days, regardless of supporting measures.
- An event meeting Hy’s Law criteria.
- Grade ≥3 non-haematologic toxicity that worsened in severity from pre-treatment baseline during the DLT-assessment period.
- Grade 3 fatigue lasting >7 days.
- In the opinion of the investigator, the AE was at least possibly related to magrolimab.

The following were exceptions to the DLT definition and not considered a DLT:

- Grade 3 anaemia.
- Grade 3 febrile neutropenia that responded clinically within 72 hours of maximal supportive care.
- Grade 3 neutropenia that resolved to grade 2 or pre-treatment/baseline with supportive care (including growth factors) within 21 days or grade 4 neutropenia lasting for ≤7 days with supportive measures.
- Grade 3 thrombocytopenia in the absence of clinically significant bleeding that resolved to grade 2 or pre-treatment/baseline within 3 weeks.
- Grade 3 indirect/unconjugated hyperbilirubinaemia that resolved to grade ≤2 with supportive care within 1 week and was not associated with other clinically significant consequences.
- Grade 3 electrolyte abnormalities that improved to grade ≤2 or baseline within 72 hours, were not clinically complicated and resolve spontaneously or responded to conventional medical interventions.
- Grade 3 elevation in alanine aminotransferase and/or aspartate aminotransferase up to 8× the upper limit of normal lasting <7 days. Grade 3 elevation in alkaline phosphatase that resolved to grade ≤2 with supportive care within 1 week and was not associated with other clinically significant consequences.
- Grade 3 nausea/vomiting or diarrhoea that resolved to grade ≤2 within 72 hours with adequate anti-emetic and other supportive care.
- Grade 3 fatigue that resolved to grade ≤2 within 1 week.
- Grade 3 infusion reactions in the absence of an optimal pre-treatment regimen.
- Grade 3 tumour lysis syndrome or electrolyte disturbances (hyperkalaemia, hypophosphataemia, hyperuricaemia) that resolved to grade ≤2 or baseline within 72 hours.
- Grade 3 hypomagnesaemia that resolved to grade ≤2 or baseline within 72 hours.
- Grade 3 or 4 lymphopenia or leukopenia not associated with other clinically significant consequences.
- Transient (≤48 hours) grade 3 local reactions, flu-like symptoms, myalgias, fever, headache, acute pain or skin toxicity that resolved to grade ≤2 within 72 hours after medical management (eg, supportive care, including immunosuppressant treatment) was initiated.
- Tumour flare phenomenon, defined as local pain, irritation or rash localised at sites of known or suspected tumour, that resolved within 72 hours with supportive care measures.
- Grade 3 lipase and/or amylase elevation without clinical or radiologic evidence of pancreatitis.

***Management of key AEs: anaemia, serious infection, and pneumonitis***

Dose modifications and/or delays were permitted to help manage AEs, including anaemia, serious infection, and pneumonitis.

To mitigate on-target anaemia associated with magrolimab treatment, patients were required to have a documented haemoglobin level ≥9 g/dL within 24 hours before each of the first 2 doses of magrolimab during initial treatment. RBC transfusions were permitted before each of the first 2 doses of magrolimab to meet this criterion. Additional haemoglobin and haematocrit checks were required 3-6 hours after initiation of the first and second doses of magrolimab during initial treatment, with RBC transfusion when clinically appropriate. Additional haemoglobin and haematocrit monitoring was recommended during the first week of magrolimab treatment in patients with symptoms of anaemia or those at increased risk for complications of anaemia.

To mitigate severe neutropenia, neutrophil counts were closely monitored, and prophylactic use of antimicrobials and administration of granulocyte-colony stimulating factor was considered when clinically indicated. Infection prophylaxis including antibiotics (eg, fluoroquinolone) or anti-fungal agents (eg, oral triazoles or parenteral echinocandin) was considered for patients with prolonged neutropenia or patients at risk. For serious infections, magrolimab dosing was withheld until clinically resolved. For serious infections that remained active for ≥14 days, discontinuation of magrolimab was considered.

If pneumonitis was suspected, inflammatory versus noninflammatory causes (eg, infections) were evaluated with imaging (eg, chest x-ray or computed tomography) and pulmonary consultation. If a noninflammatory cause was identified, the patient was treated accordingly and continued study therapy per protocol. Management of potential pneumonitis followed American Society of Clinical Oncology guidelines for immune-related AEs.^1^ Patients who experienced grade 3-4 pneumonitis were permanently discontinued from study treatment.

***Biomarker analysis***

Biomarker analysis was conducted in all patients in both SRI and randomised cohorts who received any study drug and had CD47 immunohistochemistry data available at baseline. CD47 expression was measured by EPR21794 clone on the membrane of tumour cells by immunohistochemistry. The cutoff to define CD47 low versus CD47 high (percent positive) was based on the median value of all baseline tumour samples. Disease control rate was defined as the proportion of patients who achieved a best overall response of confirmed complete response, partial response or stable disease.

***Statistical analysis***

For the randomised cohort, using an unstratified log-rank test, a total of 85 progression-free survival (PFS) events provided 73% power at a 1-sided α of 0.15 to detect a hazard ratio (HR) of 0.69 (assuming a median PFS ≥8.3 months vs a control arm median PFS of 5.7 months). Assuming an accrual period of 10 months, a minimum follow-up time of 10 months and a 5% annual dropout rate, 117 total patients (78 patients in the magrolimab arm and 39 patients in the control arm) was required to obtain 85 PFS events. Power calculations were performed using EAST 6.5. By the time of study closure, 67 patients had been randomised to the phase 2 randomised cohort, which was less than the planned sample size of 117 patients.

**References**

1. Brahmer JR, Lacchetti C, Schneider BJ, et al. Management of immune-related adverse events in patients treated with immune checkpoint inhibitor therapy: American Society of Clinical Oncology Clinical Practice Guideline. *J Clin Oncol.* 2018;36(17):1714-1768.

**Supplementary Tables**

**Table S1. Dosing and treatment schedules^a^ for all cohorts**

| **Treatment** | **Dose** | **Cycle 1** | **Cycle 2** | **Cycles 3+** |
| --- | --- | --- | --- | --- |
| **Bevacizumab^b^** | 5 mg/kg IV | Days 1, 15 | Days 1, 15 | Days 1, 15 |
| **FOLFIRI^b^** |  | Days 1, 15 | Days 1, 15 | Days 1, 15 |
| Irinotecan | 180 mg/m^2^ IV |  |  |  |
| Leucovorin^c^ | 400 mg/m^2^ IV |  |  |  |
| Fluorouracil | 400 mg/m^2^ IV bolus on first day of dose administration, followed by 2400 mg/m^2^ IV continuous over 46 hours |  |  |  |
| **Magrolimab^b,d^** | 1 mg/kg IV (priming dose) | Week 1, Day 1 | – | – |
|  | 30 mg/kg IV | Days 8, 15, 22 | Days 1, 8, 15, 22 | Days 1, 15 |

Each cycle is 28 days. All drugs were administered per standard-of-care and/or institutional guidelines.

^a^Both SRI and randomised cohorts followed the same drug dosing and schedule, as the starting dose of magrolimab for the SRI cohort and RP2D of magrolimab for the randomised cohort was 30 mg/kg.

^b^For patients receiving magrolimab in combination with bevacizumab and FOLFIRI, cycle 1, day 1 treatment could be administered over 2 days such that magrolimab was administered on cycle 1, day 1 and FOLFIRI and bevacizumab on cycle 1, day 2. This also applied to repriming cycles that required cycle 1, day 1 dosing of magrolimab. Magrolimab could be continued if combination partner drugs (bevacizumab and/or FOLFIRI) were discontinued due to unacceptable toxicity. Combination partner drugs could be continued if magrolimab was discontinued for unacceptable toxicity.

^c^Levoleucovorin 200 mg/m^2^ could be used if leucovorin was unavailable. Generics for leucovorin or levoleucovorin were also permitted. Different leucovorin doses could be used if recommended by regional or institutional guidelines.

^d^Premedication with an anti-pyretic, anti-histamine and corticosteroid was administered to mitigate IRRs. For magrolimab, this regimen was required before administration of the first 4 doses and in case of reintroduction with repriming; premedication during subsequent infusions was continued at the investigator's discretion thereafter but was mandatory if a grade 3 IRR occurred.

FOLFIRI, folinic acid (leucovorin), 5-fluorouracil and irinotecan; IRR, infusion-related reaction; IV, intravenous; RP2D, recommended phase 2 dose; SRI, safety run-in.

**Table S2. Summary of protocol deviations**

| ***n* (%)** | **Magrolimab + bevacizumab-FOLFIRI** | | | **Bevacizumab-FOLFIRI** |
| --- | --- | --- | --- | --- |
|  | **SRI cohort**  **(*n* = 10)** | **Randomised cohort**  **(*n* = 44)** | **Combined**  **(*n* = 54)** | **Randomised cohort**  **(*n* = 23)** |
| Patients with any event | 3 (30.0) | 14 (31.8) | 17 (31.5) | 4 (17.4) |
| Off-schedule procedure | 0 | 4 (9.1) | 4 (7.4) | 2 (8.7) |
| Informed consent | 0 | 2 (4.5) | 2 (3.7) | 1 (4.3) |
| Eligibility criteria | 0 | 2 (4.5) | 2 (3.7) | 1 (4.3) |
| Other treatment compliance issue^a^ | 0 | 4 (9.1) | 4 (7.4) | 1 (4.3) |
| Wrong treatment or incorrect dose | 2 (20.0) | 3 (6.8) | 5 (9.3) | 0 |
| Other^b^ | 1 (10.0) | 1 (2.3) | 2 (3.7) | 0 |
| Missing data | 0 | 1 (2.3) | 1 (1.9) | 0 |

Data presented for all enrolled patients.

^a^Category includes magrolimab priming or re-priming not done, patient received actual dose of magrolimab or FOLFIRI or bevacizumab at ≤80% of investigator-prescribed doses of study drug, and haemoglobin and haematocrit lab assessments were not obtained 3-6 hours after the first and second dose of magrolimab infusion.

^b^Category includes electronic Clinical Outcomes Assessments data transmission not completed and serious adverse event not reported to the sponsor within 24 hours of identification.

FOLFIRI, folinic acid (leucovorin), 5-fluorouracil and irinotecan; SRI, safety run-in.

**Table S3. Summary of TEAEs of clinical importance**

| ***n* (%)** | **Magrolimab + bevacizumab-FOLFIRI** | | | **Bevacizumab-FOLFIRI** |
| --- | --- | --- | --- | --- |
|  | **SRI cohort**  **(*n* = 10)** | **Randomised cohort**  **(*n* = 44)** | **Combined**  **(*n* = 54)** | **Randomised cohort**  **(*n* = 21)** |
| Number of patients with any TEAE of clinical importance | 9 (90.0) | 35 (79.5) | 44 (81.5) | 11 (52.4) |
| Anaemia^a^ | 6 (60.0) | 26 (59.1) | 32 (59.3) | 4 (19.0) |
| Infusion-related reaction^b^ | 4 (40.0) | 8 (18.2) | 12 (22.2) | 1 (4.8) |
| Pneumonitis^c^ | 0 | 3 (6.8) | 3 (5.6) | 0 |
| Serious infections^d^ | 3 (30.0) | 6 (13.6) | 9 (16.7) | 1 (4.8) |
| Severe neutropenia^e^ | 6 (60.0) | 17 (38.6) | 23 (42.6) | 7 (33.3) |
| Thromboembolic events^f^ | 1 (10.0) | 7 (15.9) | 8 (14.8) | 2 (9.5) |

AEs were coded according to Medical Dictionary for Regulatory Activities, version 27.0. Multiple AEs were counted only once per patient for the highest severity grade for each preferred term.

Data presented for all patients who received ≥1 dose of any study drug.

^a^Includes preferred terms of anaemia, blood bilirubin increased and hyperbilirubinaemia. ^b^Includes preferred terms of infusion-related reaction, dermatitis acneiform, rash, rash maculo-papular, hypersensitivity and swollen tongue. ^c^Includes preferred terms of pneumonitis and lung infiltration. No adjudication was performed for pneumonitis. ^d^Includes preferred terms of sepsis, bronchitis, COVID-19, cystitis, gastroenteritis, herpes zoster, perirectal abscess, pneumonia, skin infection, tooth abscess, urinary tract infection, vascular device infection and anal abscess. ^e^Includes preferred terms of neutropenia, neutrophil count decreased and febrile neutropenia. ^f^Includes preferred terms of deep vein thrombosis, device-related thrombosis, embolism, ischaemic stroke, pulmonary embolism, thrombosis in device, transient ischaemic attack and device occlusion.

AE, adverse event; COVID-19, coronavirus disease of 2019; FOLFIRI, folinic acid (leucovorin), 5-fluorouracil and irinotecan; SRI, safety run-in; TEAE, treatment-emergent adverse event.

**Table S4. Duration and cycles of drug exposure in treated patients**

| **Median (range)** | **Magrolimab + bevacizumab-FOLFIRI** | | | **Bevacizumab-FOLFIRI** |
| --- | --- | --- | --- | --- |
|  | **SRI cohort**  **(*n* = 10)** | **Randomised cohort**  **(*n* = 44)** | **Combined**  **(*n* = 54)** | **Randomised cohort**  **(*n* = 21)** |
| **Magrolimab** |  |  |  |  |
| Duration of exposure, weeks | 23.3 (1.1-36.1) | 14.1 (0.1-34.1) | 14.2 (0.1-36.1) | NA |
| Cycles of exposure | 5.5 (1.0-9.0) | 4.0 (1.0-9.0) | 4.0 (1.0-9.0) | NA |
| **Bevacizumab** |  |  |  |  |
| Duration of exposure, weeks | 22.6 (0.1-36.1) | 14.2 (0.1-34.1) | 14.8 (0.1-36.1) | 17.9 (0.1-34.1) |
| Cycles of exposure | 5.5 (1.0-9.0) | 4.0 (1.0-9.0) | 4.0 (1.0-9.0) | 4.0 (1.0-9.0) |
| **FOLFIRI** |  |  |  |  |
| Duration of exposure, weeks | 22.9 (0.4-36.4) | 15.1 (0.3-34.4) | 16.9 (0.3-36.4) | 18.1 (0.6-34.1) |
| Cycles of exposure | 5.5 (1.0-9.0) | 4.0 (1.0-9.0) | 4.5 (1.0-9.0) | 4.0 (1.0-9.0) |

FOLFIRI, folinic acid (leucovorin), 5-fluorouracil and irinotecan; NA, not applicable; SRI, safety run-in.

**Supplementary Figures**

**Figure S1. ELEVATE CRC CONSORT diagram.**

FOLFIRI, folinic acid (leucovorin), 5-fluorouracil and irinotecan; SRI, safety run-in.

**
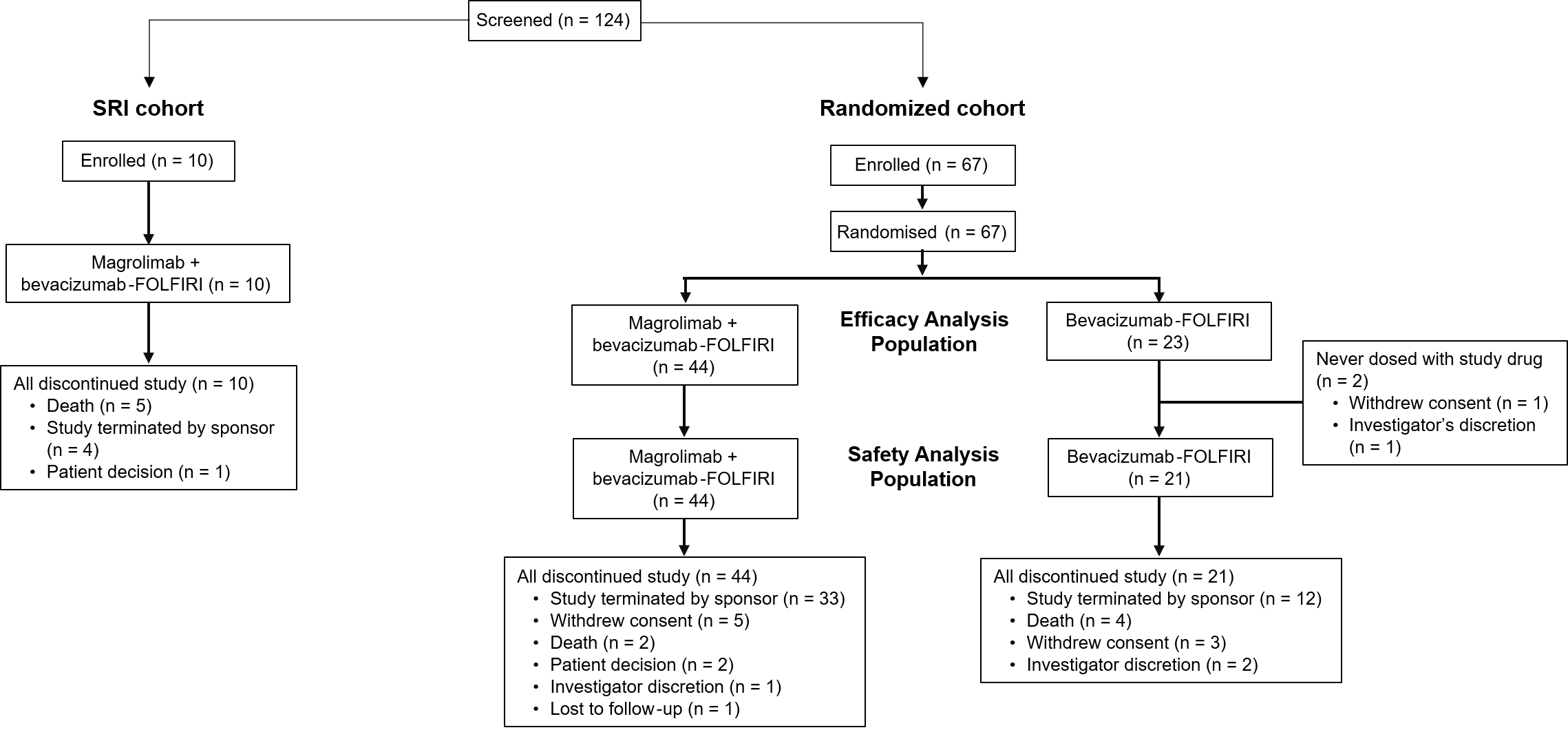
**

**Figure S2. Association between tumour cell membrane CD47 levels at baseline and response in the BEP.**

95% CI based on the Clopper–Pearson exact method.

BEP, biomarker-evaluable population; Beva-FOLFIRI, bevacizumab plus folinic acid (leucovorin), 5-fluorouracil and irinotecan; CD, cluster of differentiation; CI, confidence interval; Magro, magrolimab.

**
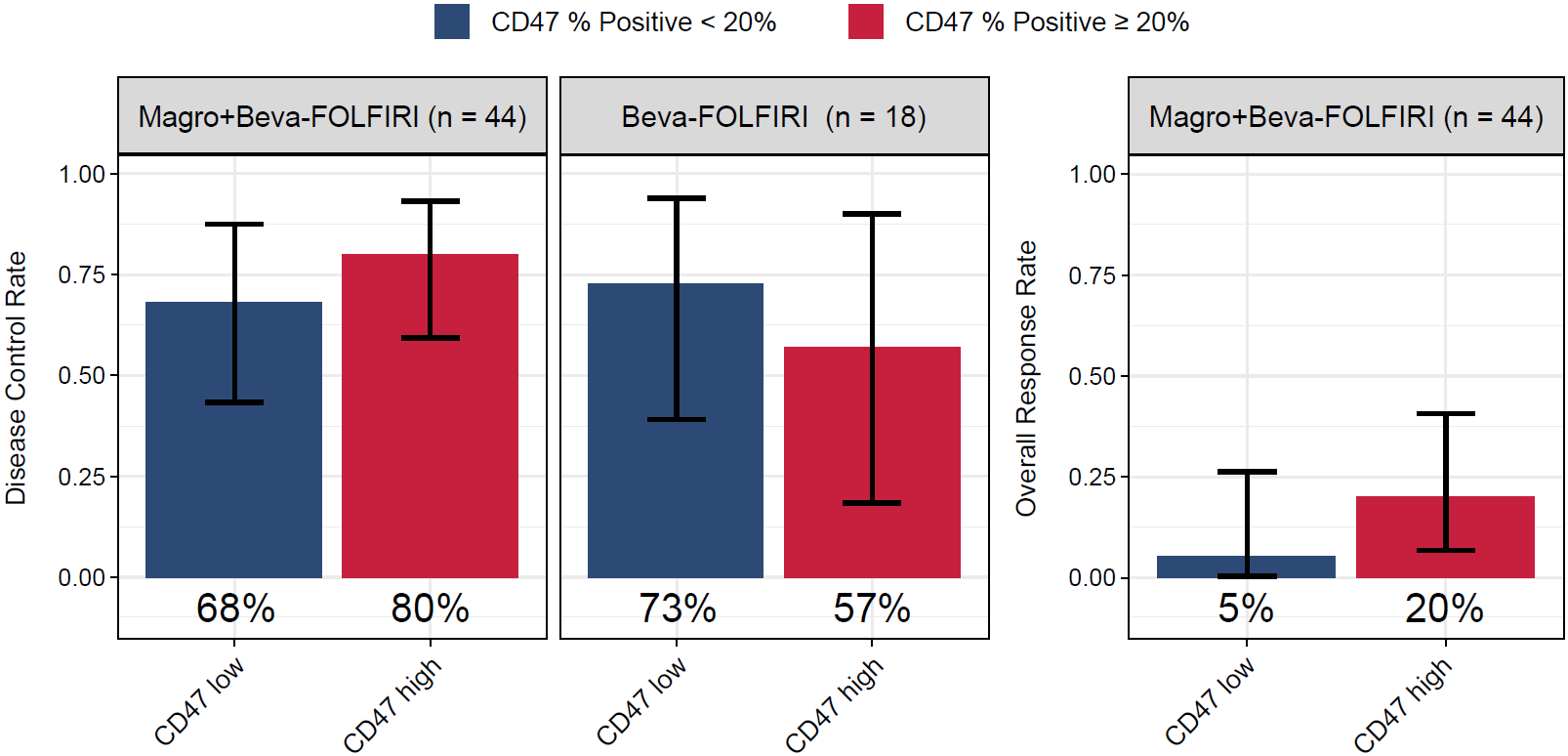
**
